# Analysis of Fiber Content and Orientation in Prefabricated Slab Elements Made of UHPFRC: Non-Destructive, Destructive, and CT Scanning Methods

**DOI:** 10.3390/ma18214843

**Published:** 2025-10-23

**Authors:** Petr Konrád, Karel Künzel, Přemysl Kheml, Michal Mára, Kristýna Carrera, Libor Beránek, Lucie Hlavůňková, Jindřich Fornůsek, Petr Konvalinka, Radoslav Sovják

**Affiliations:** 1 Experimental Centre, Faculty of Civil Engineering, Czech Technical University in Prague, Thákurova 7, 166 29 Prague, Czech Republic; petr.konrad@fsv.cvut.cz (P.K.); premysl.kheml@fsv.cvut.cz (P.K.); maramich@cvut.cz (M.M.); kristyna.carrera@fsv.cvut.cz (K.C.); jindrich.fornusek@fsv.cvut.cz (J.F.); petr.konvalinka@fsv.cvut.cz (P.K.); 2 Department of Electrotechnology, Faculty of Electrical Engineering, Czech Technical University in Prague, Technická 2, 166 27 Prague, Czech Republic; kuenzel@fel.cvut.cz; 3 Department of Machining, Process Planning and Metrology, Faculty of Mechanical Engineering, Czech Technical University in Prague, Technická 4, 160 00 Prague, Czech Republic; libor.beranek@fs.cvut.cz (L.B.); lucie.hlavunkova@fs.cvut.cz (L.H.)

**Keywords:** non-destructive testing, fiber, concrete, fiber orientation, prefabrication, CT scanning, UHPFRC, magnetism, electrical coil, quality factor

## Abstract

This study investigates fiber content and orientation in prefabricated slab elements made of ultra-high-performance fiber-reinforced concrete (UHPFRC), using novel non-destructive measurement using a coil’s quality factor, where the coil is put to one side of the specimen only. This allows the analysis of slab specimens of arbitrary size. That then allows an accurate quality control of elements made in the prefabrication industry. This study presents an experimental campaign designed to evaluate the non-destructive principle’s accuracy and practical feasibility. Twenty-five large slab specimens were made in an industrial prefabrication plant using various casting methods to introduce different flow-induced fiber parameters. The slabs were subjected to this non-destructive testing, then destructive bending tests and CT scanning to tie the results together and validate the non-destructive results. The results showed that the coil’s quality factor values correspond well to the distribution (concentration) and orientation of fibers, and the method reliably reveals potential defects of the material and can predict the element’s mechanical properties.

## 1. Introduction

Fiber-reinforced concrete has significantly increased in popularity in various civil engineering projects in the last decades, ranging from factory floors to bridge decks [1,2,3,4]. The material mitigates the inherent mechanical problems of ordinary concrete—its brittleness. Traditional reinforced concrete deals with concrete’s lack of tensile strength; however, it does not solve the problem of small crack formation and propagation. Fibers, especially small but numerous fibers in high-performance concretes, can effectively control the cracks and, together with the matrix, form a ductile composite [5]. However, the major problem with fiber reinforcement is the uncertain final mechanical behavior, as fibers are randomly distributed and oriented in the volume. The mixture needs to be designed well so that fibers will not exhibit separation or sinking. During mixing, fibers must be carefully introduced so as not to form clusters. Placement of fresh concrete can also introduce strong preferential orientation [6,7]. All of these steps can potentially introduce negative consequences regarding fiber distribution and orientation that could unacceptably influence the final mechanical properties [8,9,10,11]. It is possible to check the fiber reinforcement destructively using tensile (or flexural) strength tests. Another possibility is to slice the elements and conduct visual analysis of the cut fibers, which is a method used frequently in laboratory studies, usually to correlate with other parameters [12,13,14]. Small specimens can also be cut from the element to subject them to X-ray imaging methods [11,15]. However, these methods cannot be used to check each manufactured element. A truly non-destructive method is required for that purpose.

In the literature, in the last 25 years, numerous methods of estimating fiber parameters have been developed, which only highlights the motivation to come up with a reliable and practical non-destructive solution. Examples of methods include measuring electrical impedance between two points on the specimen [16,17]. This method presents several disadvantages. The most severe is the influence of the concrete matrix itself, where its own electrical parameters play a role. After all, measuring such parameters is the basis of non-destructive monitoring of concrete’s age [18]. Microwave methods have been studied as well [19], but the problem of separating concrete and fiber parameters is present, too. This is specifically highlighted by different results for dry and wet concrete. Similar problems and relatively low sensitivity to fiber parameters can be expected when using ultrasound methods [20]. Additionally, all of these methods require attaching probes to the specimen, where the quality of contact plays a key role. Some of these disadvantages are also reported by Cavalaro et al. [21].

In more recent studies, focus has shifted towards magnetic-based methods. Here, the magnetic properties of an electrical coil would be measured. If a ferromagnetic material, like a steel fiber, comes close to the coil, its parameters change. This method completely removes the influence of the concrete matrix, removes the need to carefully attach probes, and even has the potential for higher accuracy and sensitivity to fiber parameters. However, the approaches to measuring the coil and even the coil’s construction vary between studies. The most frequently found is the impedance principle—measuring the electrical impedance of the coil. One of the earliest practical studies on this topic was made by Torrents et al. [22]. In this study, the authors used a large electrical coil that would fit an entire 150 mm concrete cube inside. They confirmed the feasibility of this approach and even confirmed that the concrete age does not influence the measurements. However, such an electrical coil is not quite practical for other geometries or full-scale concrete products. Cavalaro et al. [21] then improved the interpretation of these results and provided more detailed theoretical explanations of the measured phenomena. Faifer et al. [23] then experimented with a coil wound over a C-shaped ferrite core, which they used to conduct measurements on one surface only. However, the coil lacked the required sensitivity. An improved double coil (excitation coil and measurement coil) was used next in their study, which showed improvement at the expense of simplicity. Similar excitation-pickup coil setup was used by Frankowski et al. [24] to detect traditional reinforcing bars. Further studies can be found in the literature that focused on inductive measurement, even in recent years, highlighting how work in the area is still necessary and ongoing to find the optimal approach [25,26,27,28], while also using other methods to confirm the accuracy [29].

A similar approach of using a measuring coil was also used by our research team during ongoing research related to the magnetic orientation of fibers [30]. However, the focused quantity was the coil’s quality factor, which was found to be more sensitive to fiber parameters. This requires a construction of measuring coils specific to this measured quantity, which is also the main novelty. This paper uses a newly developed surface measuring apparatus—a quality factor measuring coil that will be put on one of the surfaces only, measuring the specimen from the top and from below. Using this new apparatus is one part of the presented research, together with confirmations from destructive bending experiments and CT scans.

## 2. Materials and Methods

### 2.1. Prefabricated Slabs

**Figure 1:**
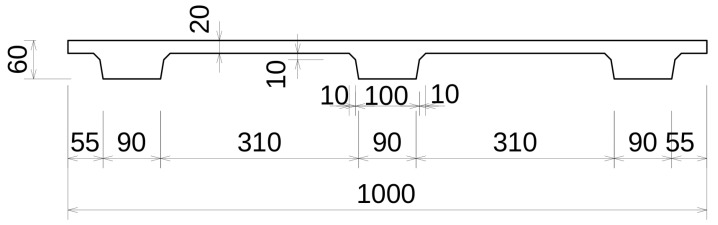
Cross-section of the slab specimens.

The slab specimens used in this study were manufactured in a commercial prefabrication company (KŠ Prefa, Prague, Czech Republic) and delivered to the laboratory for all of the experiments. The specimen’s cross-section can be seen in Figure 1, and it consisted of a slab stiffened by three ribs. Its length was 1470 mm. The material used was ultra-high-performance fiber-reinforced concrete (UHPFRC). Its specified type is C120-FR7-B-XF2-CI 0.2-Dmax 2-KV-VS10 according to the Czech technical requirement TP 267 Ultra high-performance concrete [31] and the European norm EN 206 (its Czech version ČSN EN 206+A2: Concrete—Specification, performance, production, and conformity [32]). Strength class is 120 MPa. Mixture details are given in Table 1. As it is a commercial product, further information regarding the concrete’s constituents was not disclosed. Fibers used were straight steel with dimensions of 14.0 mm × 0.2 mm and a strength of 2 GPa. Their amount was 1.5% of the mix volume.

**Figure 2:**
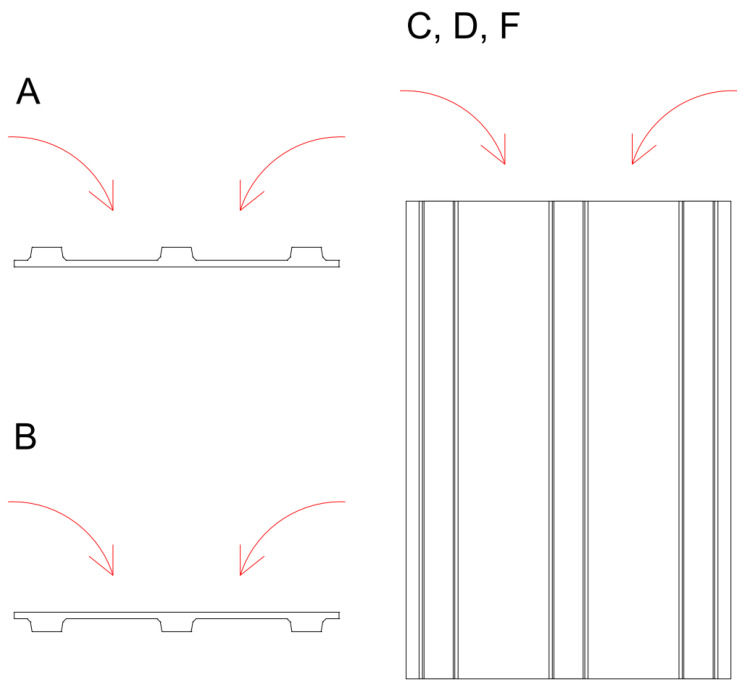
Directions of pouring different series of specimens.

In total, 25 specimens were used, 5 specimens in 5 series (A, B, C, D, and F—series E was used in unrelated research). Each series differed only in the method of placing fresh concrete into the molds. Figure 2 highlights the differences. Series A was cast with the mold lying flat with the ribs facing up. Series B reversed, with ribs facing down. Series C, D, and F were cast with the mold in a vertical position in separate pouring sessions for each series. Series C had concrete poured out of the industrial hopper in the area of the center rib, so the material filled the rest of the volume on its own. Series D was filled uniformly along the entire filling opening of the mold. These four series were prototype productions cast in wooden molds; series F specimens were taken from a subsequent commercial series production using steel molds but were otherwise poured the same as series D. Vibration was not used to prevent sinking of fibers.

### 2.2. Non-Destructive Coil Quality Factor Measurements

**Figure 3:**
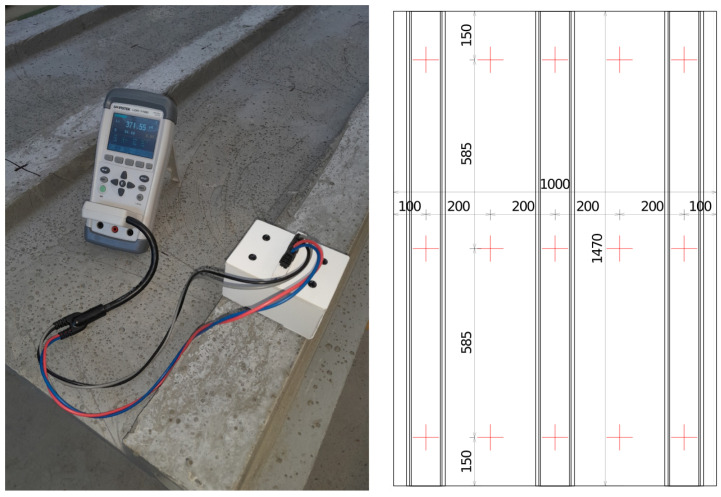
(**Left**)—the measuring device on top of the slab specimen. (**Right**)—the measuring pattern, with a cross indicating the two measured axes for each point. Dimensions in mm.

The non-destructive testing for evaluating the fiber parameters in hardened concrete utilized the quality factor measurements of an electrical coil. A novel device was used for this purpose that can be practically employed in industrial environments. Since large slab specimens were to be tested, the relatively older coil principle [30], where the entire specimen needs to fit inside the coil, could not have been used. This new device uses a coil wound over a half-toroidal core and can be placed to the side of the specimen, where the nearby fibers change the coil’s parameters (Figure 3, left). A portable LCR meter (GW Instek LCR-1100, GOOD WILL INSTRUMENT CO., LTD., New Taipei City, Taiwan) is used to acquire the data and directly send it to a data-logging laptop, where an application was developed for automatically formatting the data based on the measuring pattern. The measuring pattern can be seen in Figure 3, right. A portable and inexpensive LCR meter was chosen specifically to showcase how the method does not require a highly precise laboratory desktop LCR meter. The portable meter’s measuring frequency is 100 kHz, which is also conveniently a frequency that was identified as suitable for coil fiber measurements in the authors’ previous research [30,33]. The meter uses an equivalent series circuit to determine the quality factor. Test speed was set to “slow”—2.3 s^−1^—and the value was recorded approximately 2 s after placing the coil, when the values stabilized. Range was set to automatic. The estimated penetration depth of the meter is 10 mm for these specific conditions. This highly depends on fiber volume and orientation itself. The coil was made using four identical half-toroid K10 cores (TDK, Tokyo, Japan), each with an outer diameter of 87 mm, an inner diameter of 54.3 mm, and a thickness of 13.5 mm. A frame made of foamed PVC was built around it, and 50 turns of high-frequency stranded wire were wound. This wire consisted of 120 conductors, each 0.1 mm in diameter, braided with silk insulation. The coil inside a 3D-printed housing was always placed directly on the concrete surface to maintain the same lift-off. The measured area corresponds to the footprint of the coil, so approximately 80 mm × 40 mm. Further extensive information is provided in the authors’ other publications—background of the measuring principle, detailed construction of the meter and its laboratory testing [34]; measuring of fiber properties, theory of fiber detection, and experimental validation [30]; numerical simulations; and theoretical and experimental behavior of fibers in a magnetic field [35].

Both sides of the slab were measured in the same planar positions, and each point was measured in two axes (parallel to the longer and shorter sides of the slab), as indicated by the crosses in Figure 3, right. The measuring pattern was chosen to have at least 5 points in one row to measure all 3 ribs and the 2 thin areas in between. At least 3 rows were needed to assess both ends, where fibers were likely to either concentrate or exhibit deficiency (bottom and top of the mold for manufacturing methods C, D, and F), and the middle of the specimen, which would be crucial to bending performance. Thus, 5 points per row times 3 rows times 2 sides times 2 axes for each point equals 60 total measurements per specimen. Each specimen was measured once. Measuring in both axes reveals information regarding the fiber orientation, as the two measurements differ. This is further explained together with the experimental results in Section 3. The theoretical basis for interpreting the measured values is also further explained in references provided above in the previous paragraph.

### 2.3. Destructive Bending Experiments Setup

**Figure 4:**
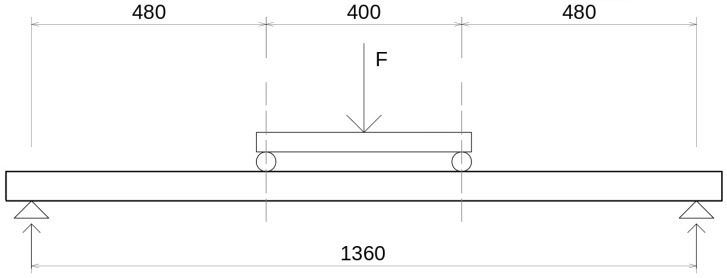
Schematic of the bending experiment.

After the non-destructive evaluation, all specimens were subjected to destructive bending experiments. The setup was a four-point bending experiment with a 400 mm span between loading bars and a 1360 mm total span, as seen in Figure 4. The slabs were positioned with the ribs facing up, as that is the intended position of the product acting as a stay-in-place formwork. The load was force-controlled with unloading cycles at specific forces, i.e., the loading regime was 0 kN–4.5 kN–0 kN–9 kN–0 kN–15 kN–0 kN—up to total breakage. The cycles represent different phases at the construction site—placing the slab, placing reinforcing bars on top, and pouring concrete. The loading speed was 5 kN·${min}^{-1}$ for the cycle phase and 10 kN·${min}^{-1}$ in the last loading phase. Two potentiometer displacement meters were acquiring the deformation in the center of the span on the sides of the specimen. Their measurements were averaged.

### 2.4. CT Fiber Scanning

After the destructive experiments, several specimens were prepared for X-ray CT scanning analysis to see the fiber concentration and orientation precisely. The CT analysis was possible to be done on specimens with a maximum size of 120 mm × 180 mm, so this size was cut, using a diamond saw, from the slabs. These cutouts were done from positions where the quality factor was measured. Due to the very large total volume of concrete subjected to testing, only selected slabs and selected positions from the slabs were chosen to be cut and prepared. Positions in the middle of the slabs were damaged by the flexural tests, so those were not used. Other positions were selected with the intent to capture various fiber phenomena due to the manufacturing methods and see if the CT images corresponded to measured quality factors. The CT scanner used was Zeiss Metrotom 1500 (ZEISS group, Oberkochen, Germany). Specimens cut only at the slab (total thickness 20 mm) were scanned using the following settings: prefilter Cu 2.00 mm, 1150 projections, dynamic correction for each projection, enabled scattered radiation correction, 190 kV X-ray tube voltage and 957 μA current, total scanning time 44 min. Specimens cut at the rib area (total thickness 60 mm) used settings—prefilter Cu 3.00 mm, 1450 projections, dynamic correction for each projection, enabled scattered radiation correction, 220 kV X-ray tube voltage and 1286 μA current, total scanning time 54 min. Both situations had 2 s integration time, gain 16×, no image averaging, 1 × 1 binning, and 1 × 1 hardware binning. Image processing was done in VG Studio MAX 3.2.2 using the Fiber Composite Material Analysis. The analysis identified fibers and assigned colors to them based on their orientation for better visualization.

## 3. Results and Discussion

### 3.1. Destructive Bending Tests

Table 2 shows the results of the destructive testing. Flexural strength was calculated using the following formula:

(1) $$\sigma =\frac{0.5Faz}{I}$$

where *F* is the loading force, *a* is the distance between the support and the closer loading cylinder (480 mm), *z* is the distance between the cross-section’s centroid and the lowest point of the cross-section (39.16 mm), and *I* is the cross-section’s moment of inertia (8,620,422.55 mm^4^).

The results show a clear influence of the casting direction. Series A exhibited the highest flexural strengths, as it was manufactured in a way that caused the material to achieve the highest quality in the flat part, i.e., the tension part. The performance was also likely helped by the slight sinking and, therefore, the concentration of fibers into the tension area. Series B, on the other hand, was manufactured the opposite way, so a decrease in strength is logical. Three specimens of series C were the only ones that did not even reach the third peak of the loading cycle of 15 kN, so it was not possible to completely finish the loading regime. This series, therefore, failed, as it did not meet the design criteria. This was a direct result of the pouring method, where fresh concrete was poured only into the central rib, letting the concrete flow to the rest of the volume, but this must have caused fiber separation. Series D and F removed this effect by evenly pouring concrete into the entire cross-section.

**Figure 5:**
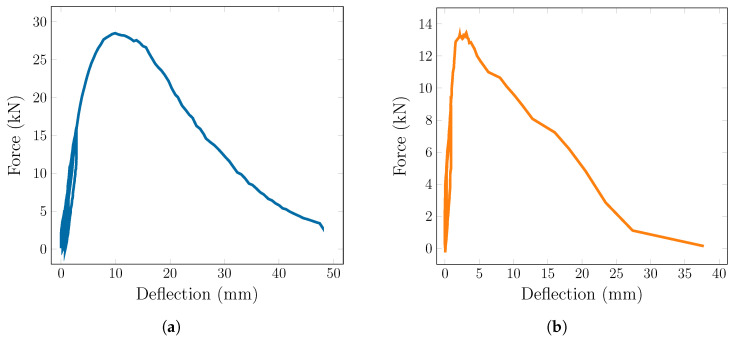
Examples of load-displacement diagrams: (**a**) specimen 1A (**b**) specimen 2C.

Figure 5a shows an example of a load-displacement diagram of specimen 1A, as it is representative of all specimens in series A, B, D, and F. The loading cycles can be seen at the beginning of the chart, mostly in a linear regime with limited plastic deformation. Before reaching the peak force, there is a region of deflection hardening, followed by pronounced deflection softening as fibers are being pulled out of the matrix in propagating main cracks. Figure 5b shows the load-displacement diagram of specimen 2C, which is an example of a situation when the specimen failed before 15 kN. It can be seen that the deflection hardening region is negligible and the specimen undergoes a more brittle crack, followed by limited fiber-bridging behavior, indicating a problem with either fiber voids or strong orientation parallel to the crack. The rest of the load-displacement diagrams follow very similar shapes.

### 3.2. Non-Destructive Quality Factor Measurements

The non-destructive quality factor measurement results are in line with the destructive results and explain the observed behavior. Two parameters were measured—fiber concentration and fiber orientation. This was achieved by measuring one point in two axes, as explained in Section 2.2. If we average the two measurements, so a single value is presented for a point, then a heatmap can be presented over the specimen’s plane, where the values should ideally be all identical for perfect fiber distribution. It must be noted that a single value on its own is largely irrelevant, as it is a comparative measurement, so comparison across the specimen is required, hence the heatmap. Values in the heatmaps higher than others indicate fiber deficiency at that point; values lower than others reveal fiber concentration. Next, a ratio can be made of the two measured values for a single point, which shows information regarding the orientation. In that case, values higher than 1.0 point to preferential orientation parallel to the specimen’s ribs, and values lower than 1.0 perpendicular to the ribs.

**Figure 6:**
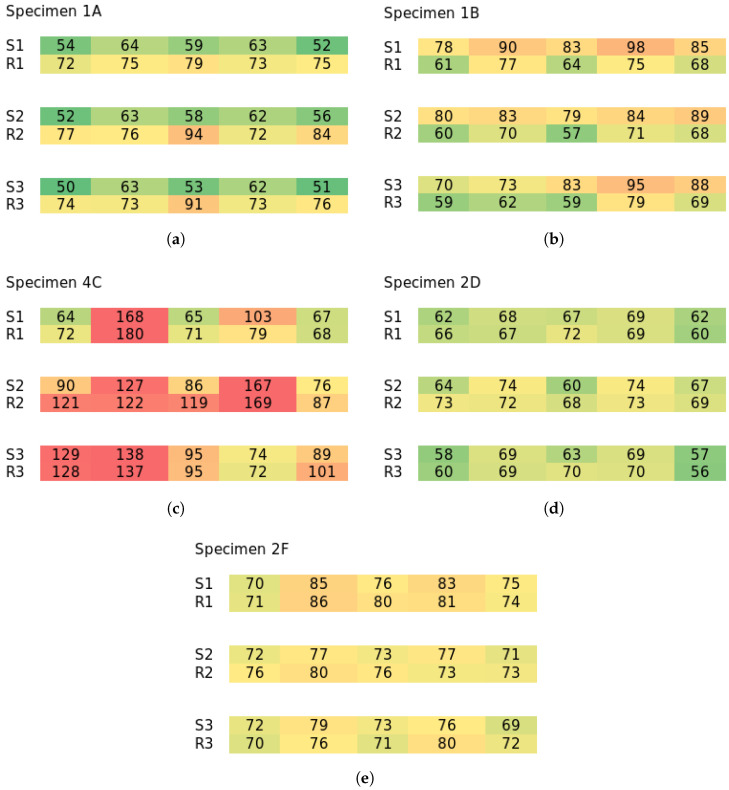
Selected results of fiber concentration analysis. The rest of the results in Appendix A. Colors are the heatmap of values for one specimen. (**a**) Specimen 1A; (**b**) specimen 1B; (**c**) specimen 4C; (**d**) specimen 2D; (**e**) specimen 2F.

Figure 6 shows examples of selected specimens that exhibited noteworthy results regarding the fiber concentration (the rest of the results are in). Row S1 is measurements on the smooth side of the specimen in the first row (see Figure 3, right). Row R1 is measurements on the ribbed side opposite of measurements S1. Rows 2 and 3 are formatted the same. This means that Figure 6a, showing specimen 1A, points to moderate fiber concentration in the slab, while especially the center rib shows fiber deficiency. This would be in line with the destructive testing, as fibers were, therefore, more present in the bottom part of the specimen undergoing tension (ribs were facing up during bending). The example of specimen 1B shows the opposite measurements, which are also in line with the bending performance. Series C showed the highest differences in measured values, pointing to major fiber concentrations and voids. As expected, the outer parts of the specimens were lacking fibers. Series D and F have fibers mostly uniformly distributed; the example in Figure 6d shows specimen 3F, where the heatmap is very uniform. For better readability, the rest of the specimen’s measurements are placed in Appendix A.

**Figure 7:**
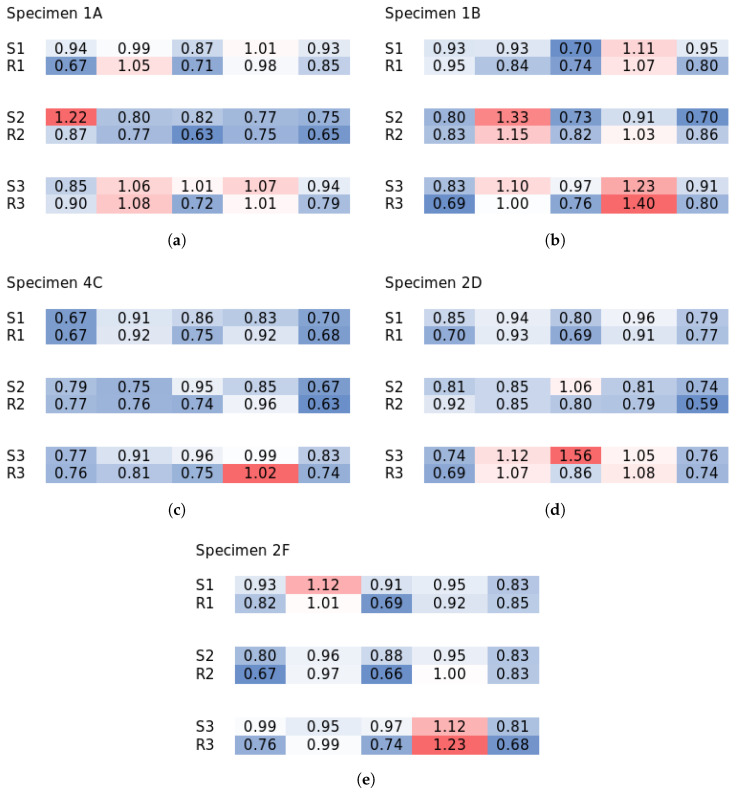
Selected results of fiber orientation analysis. The rest of the results in Appendix B. Colors highlight deviations from 1.0. (**a**) Specimen 1A; (**b**) specimen 1B; (**c**) specimen 4C; (**d**) specimen 2D; (**e**) specimen 2F.

Figure 7 shows the same specimens as for the concentration examples, but for the orientation ratios (the rest of the results are in Appendix B). Specimen 1A also exhibited moderate preferential orientation in the middle of its longitudinal span parallel to the ribs, which would enhance its flexural performance in the given direction. That is what was obtained from the destructive tests. Series B, on the other hand, showed stronger preferential orientation in the slab between the ribs perpendicular to the ribs, while ribs were oriented parallel, which is once again in line with the destructive testing. Series C overall showed moderate orientation parallel to the ribs in the whole volume, but these results clearly point to the fact that the orientation results alone do not predict the behavior well, as the concentration information is perhaps even more vital. Series D and F then repeated the trend of series B, but with not as pronounced differences. The orientation in specimens must have been the result of the casting direction, as fibers would be oriented by the fresh concrete’s flow. The vertically poured specimens would then be the most influenced by flow, where the 60 mm thick rib volume would act more like a tube. Fibers there would orient parallel to the flow, and some fibers could also be oriented along the thickness, which would not be revealed by the orientation analysis. On the other hand, in the relatively thin 20 mm slab volume, fibers likely exhibited the planar orientation perpendicular to the flow direction, which would be in line with the study conducted by Boulekbache et al. [36].

### 3.3. Correlation Analysis—Flexural Strength and Quality Factor

All of the obtained quality factor values were analyzed for correlation with flexural strength to reveal how the quality factor could predict flexural strength, i.e., how reliable it is as a means of quality control. Several values were prepared for each specimen—averages of both concentration and orientation for all measurements, only on the top (ribbed side), only on the bottom (smooth side), ratios of bottom to top, only on the top in the middle span, only on the bottom in the middle span, and the ratio of bottom to top only in the middle span, plus standard deviations for all.

The best correlation to strength was observed for the concentration measurements when only taking into consideration the average of the five concentration values obtained for the bottom of the specimen in the middle of the span. The correlation coefficient was −0.76 in that case. This is logical, as due to the setup of the bending test, that area is where the highest stress is present, so fiber parameters are key there. On the other hand, the average of the five concentration values on the top of the specimen in the middle of the span has a correlation coefficient of −0.62, indicating how the top surface concentration is less relevant to flexural strength. The orientation values were overall poorly correlated with strength. For the middle of the span, only at the bottom, the correlation coefficient was −0.21. This proves that the orientation data alone are meaningless if the concentration (or generally fiber volume) parameters are unknown.

With these assumptions in mind, a prediction model can be formulated. Only the middle span bottom values are used from now on. The input assumptions are that average concentration is key, its standard deviation is largely collinear, but it points to concentration inhomogeneity, so it should be included; orientation effects need to be applied based on concentration level, as fewer fibers mean lesser orientation influence; similarly, standard deviation of orientation should be included, as it points to inhomogeneity; and orientation is likely related non-linearly. The model can be written as

(2) $$F_{predict}=a_{0}+a_{1}C+a_{2}C_{stdev}+\frac{b_{1}\left(c_{1}O+c_{2}O_{stdev}\right)+b_{2}{\left(c_{1}O+c_{2}O_{stdev}\right)}^{2}}{1+d_{1}C+d_{2}C_{stdev}}$$

where $a_{i};b_{i};c_{i};d_{i}$ are fitting coefficients (weights), *C* is the average of concentration, $C_{stdev}$ is the standard deviation of *C*, *O* is the average of orientation, and $O_{stdev}$ is the standard deviation of *O*. Fitting the data using the non-linear least squares method, the coefficients equal to

$$\begin{array}{c} a_{0}=36.710\\ a_{1}=-0.524\\ a_{3}=0.726\\ b_{1}=9.594\\ b_{2}=-1.064\\ c_{1}=5.542\\ c_{2}=7.227\\ d_{1}=-0.008\\ d_{2}=0.062 \end{array}$$

**Figure 8:**
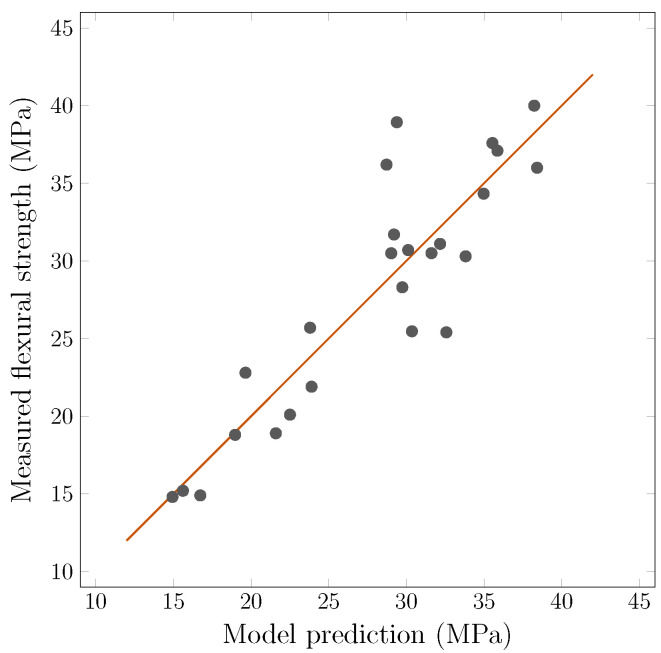
Model based on the coil’s quality factor to predict the flexural strengths with an ideal line. Specimens above the line would have their strengths underestimated.

These values point to an overall relatively small influence of orientation. This is likely because the orientation in this data set did not drastically vary, as opposed to the concentration. The final correlation coefficient for this model is 0.89. Figure 8 shows a plot of the measured strengths to the model’s output with an ideal fit line. Other models were tested with simplified parameters but showed worse correlation. The number of measured quality factor points on a specimen was chosen to be minimalistic to have a basic overview of the slabs’ manufactured quality. The results showed that for a more accurate prediction of strength, further points would be needed, especially in the entire area of maximum stresses. It should also be noted that the number of specimens, while not low, could be higher to improve the statistical value.

### 3.4. CT Scanner Images

**Figure 9:**
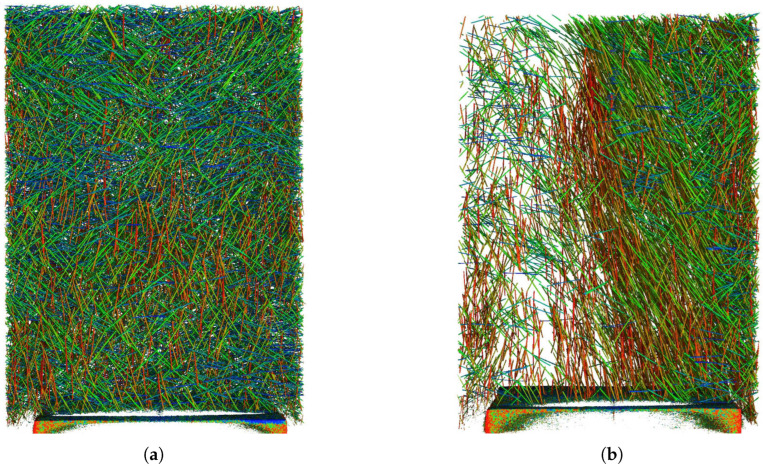
CT scanner analysis, cutout from specimens 1B (**a**) and 2C (**b**). Colors represent different fiber orientation.

The CT scanner analysis confirmed the previous measurements and allowed a detailed view into the specific fiber orientation. The following images were selected, as they show interesting fiber phenomena. The rest of the obtained images are in Appendix C with indicated positions in the specimen. Figure 9a shows specimen 1B in the measurement area of the first row, second column (see Figure 3). This image overall shows an ideal situation regarding expected fiber behavior. Distribution inside the scanned area was roughly uniform. The quality factor values indicated minor preferential orientation in the specimen’s longitudinal direction, which can be observed here due to the slight prevalence of the red-colored vertically aligned fibers. No flow-induced preferential orientation is visible, as these specimens were cast lying flat on the ground, not creating the opportunity for the fresh concrete to flow far. On the other hand, Figure 9b shows a defect in the measurement area of the first row, second column of specimen 2C. A major deficiency of fibers with a clear dividing line can be seen. The edge of the denser area is strongly oriented parallel to this edge, which likely points to a flow-induced orientation in the image from right to left and subsequent separation of the concrete matrix with a decreased amount of fibers. The quality factor analysis already showed a possible fiber deficiency; however, as the measurement was done right at this density-dividing edge, the values were not as strong, suggesting a major failure. This leads to a possible recommendation that the non-destructive analysis could be done at more points. The oriented edge of the denser area was not revealed with the orientation analysis, as the edge was rather thin, and the rest of the volume under the quality factor meter was oriented more randomly. The lowered density of fibers also lowers the method’s sensitivity to orientation.

**Figure 10:**
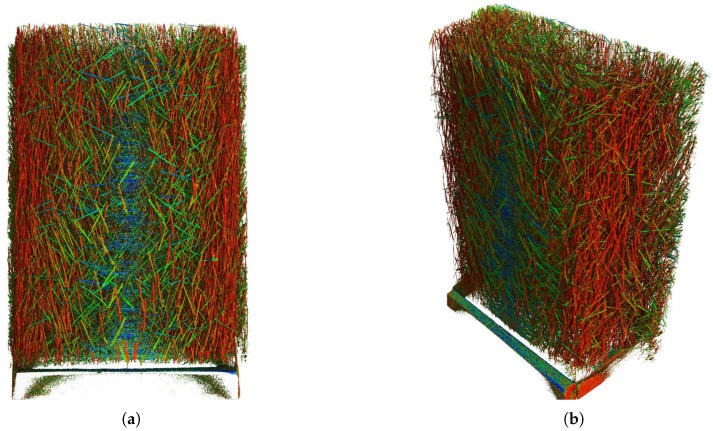
CT scanner analysis, cutout from specimen 5D front view (**a**) and angled view (**b**). Colors represent different fiber orientation.

Figure 10 shows the first row, third column of specimen 5D, therefore, in the rib area. The non-destructive concentration measurement showed mostly optimal distribution of fibers throughout the specimen. The orientation analysis pointed to a moderate preferential orientation in the specimen’s longitudinal direction. This is apparent in all specimens, but most strongly in the vertically cast series C, D, and F. The strongly oriented fibers are located towards the walls of the rib, which is the result of the wall effect, where fibers align with the wall in the direction of the concrete’s flow. Interestingly, this effect is practically not visible on the wider, central wall of the rib, where fibers are oriented more perpendicularly. Nevertheless, the entire scanned specimen shows, on average, a prevalent red color, which again confirms the non-destructive analysis.

## 4. Conclusions

This presented study showed the feasibility of using the portable non-destructive quality factor measurement principle for analyzing large prefabricated elements. The results were in line with the destructive test and CT scans. The specific points of the research and subsequent conclusions can be summarized as follows:

- The quality factor measurements accurately evaluated fiber concentration and orientation, so the method can be used as a quality control principle in the prefabrication industry.
- The method is relatively simple and inexpensive to apply for quick quality control. However, more complex solutions (integrating into an automated production line, continuous scanning, finer scanning mesh, or adaptive measurement point selection) could be possible future headings if more detailed measuring data are desired.
- Both are comparative measurements. Fiber concentration values should be similar throughout the specimen.
- Fiber orientation is strongly dependent on the casting direction, as expected, and the quality factor values confirmed it.
- Measured quality factor values can be used to predict the overall mechanical behavior, especially in regard to identifying major material defects.

## Figures and Tables

**Table 1:** Mixture constituents.

Constituent	Part
Cement	36% weight
Sand 0/2 mm	59% weight
Slag, ash, silica fume	11.5% weight
Admixtures	3.5% weight
Steel fibers	1.5% volume

**Table 2:** Results of the bending experiments. Values in brackets are standard deviations.

Specimen	Maximum Force (kN)	Flexural Strength (MPa)	Average Flexural Strength (MPa)
1A	28.52	31.1	34.9 (4.1)
2A	27.82	30.3	
3A	32.99	36.0	
4A	36.67	40.0	
5A	34.07	37.1	
1B	20.05	21.9	22.1 (3.4)
2B	17.22	18.8	
3B	17.35	18.9	
4B	23.28	25.4	
5B	23.54	25.7	
1C	18.43	20.1	17.5 (3.7)
2C	13.62	14.9	
3C	13.55	14.8	
4C	13.95	15.2	
5C	20.90	22.8	
1D	27.94	30.5	31.5 (2.9)
2D	28.12	30.7	
3D	33.19	36.2	
4D	29.09	31.7	
5D	25.95	28.3	
1F	31.49	34.3	33.4 (5.5)
2F	34.48	37.6	
3F	35.71	38.9	
4F	27.97	30.5	
5F	23.36	25.5	

**Figure A1:**
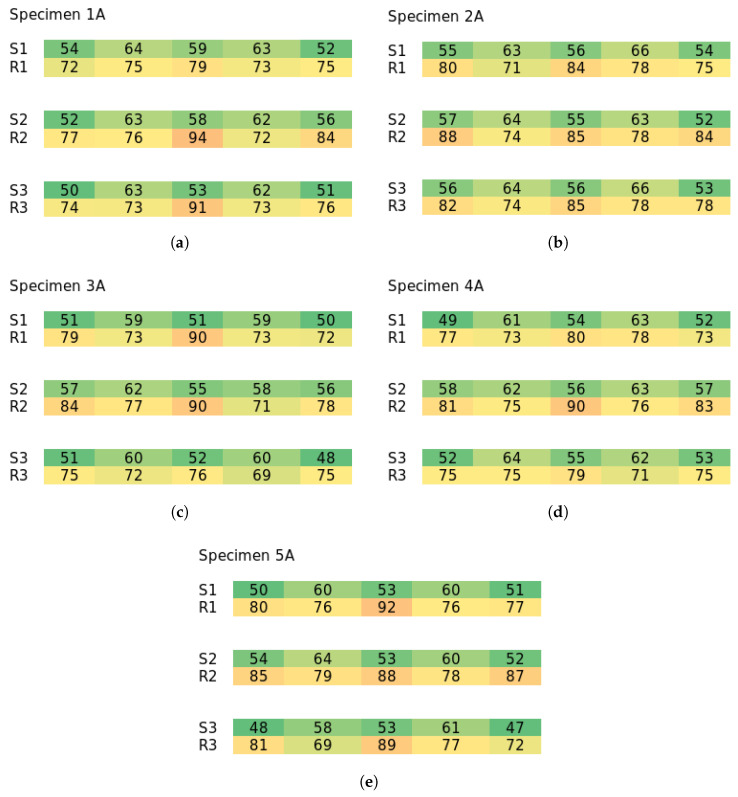
Results of the non-destructive concentration analysis, series A. Colors are the heatmap of values for one specimen. (**a**) Specimen 1A; (**b**) specimen 2A; (**c**) specimen 3A; (**d**) specimen 4A; (**e**) specimen 5A.

**Figure A2:**
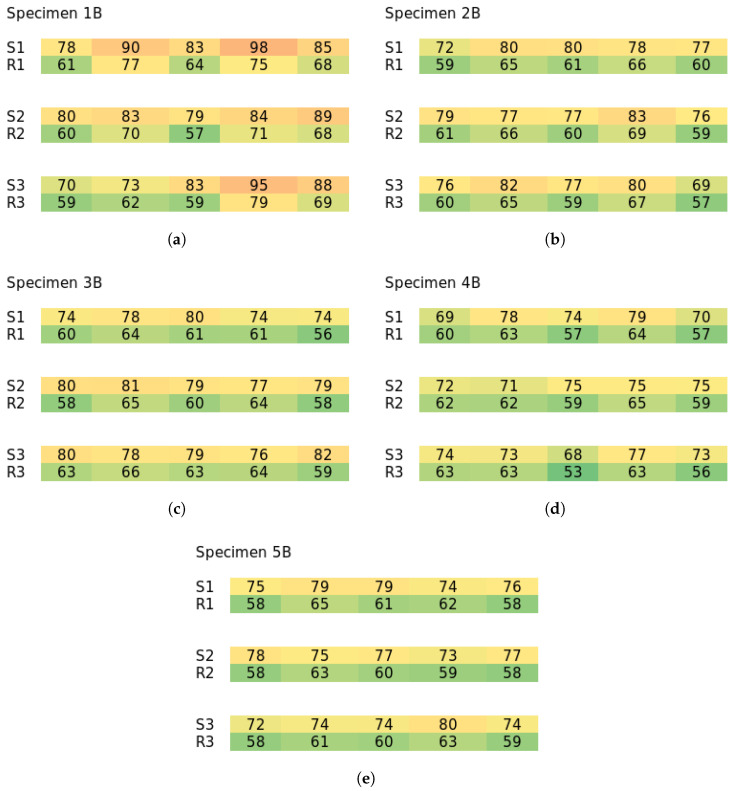
Results of the non-destructive concentration analysis, series B. Colors are the heatmap of values for one specimen. (**a**) Specimen 1B; (**b**) specimen 2B; (**c**) specimen 3B; (**d**) specimen 4B; (**e**) specimen 5B.

**Figure A3:**
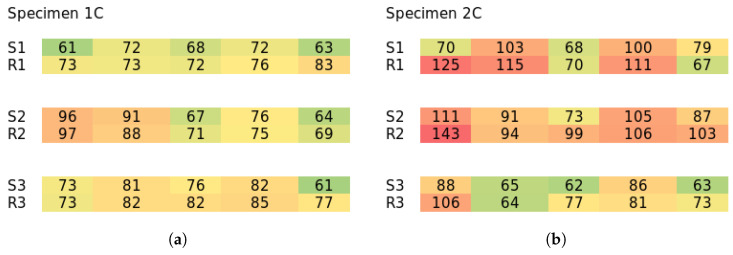

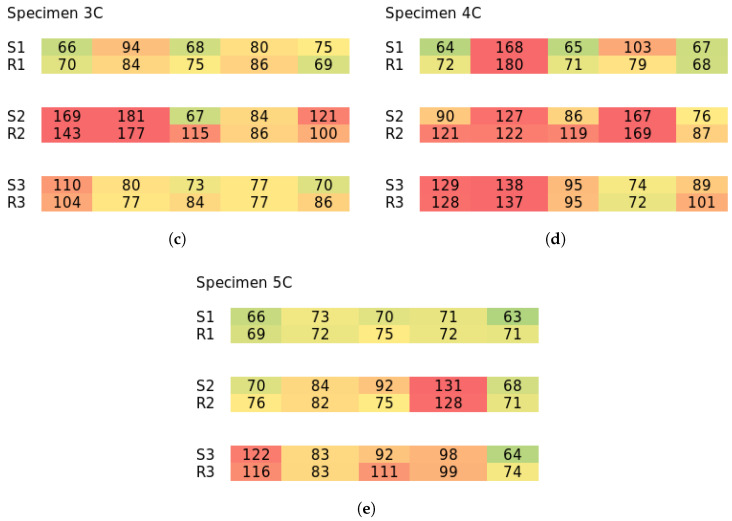
Results of the non-destructive concentration analysis, series C. Colors are the heatmap of values for one specimen. (**a**) Specimen 1C; (**b**) specimen 2C; (**c**) specimen 3C; (**d**) specimen 4C; (**e**) specimen 5C.

**Figure A4:**
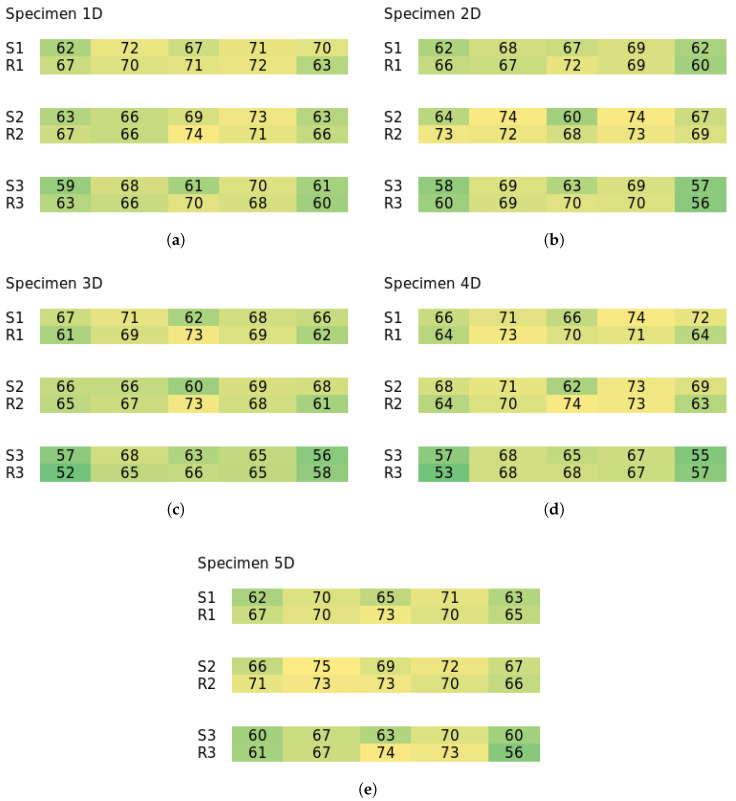
Results of the non-destructive concentration analysis, series D. Colors are the heatmap of values for one specimen. (**a**) Specimen 1D; (**b**) specimen 2D; (**c**) specimen 3D; (**d**) specimen 4D; (**e**) specimen 5D.

**Figure A5:**
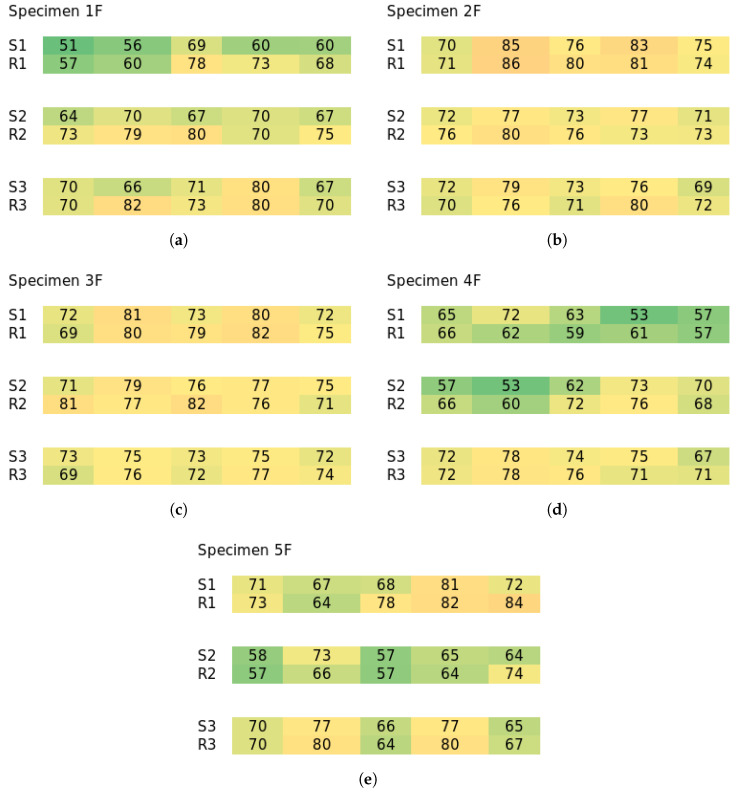
Results of the non-destructive concentration analysis, series F. Colors are the heatmap of values for one specimen. (**a**) Specimen 1F; (**b**) specimen 2F; (**c**) specimen 3F; (**d**) specimen 4F; (**e**) specimen 5F.

**Figure A6:**
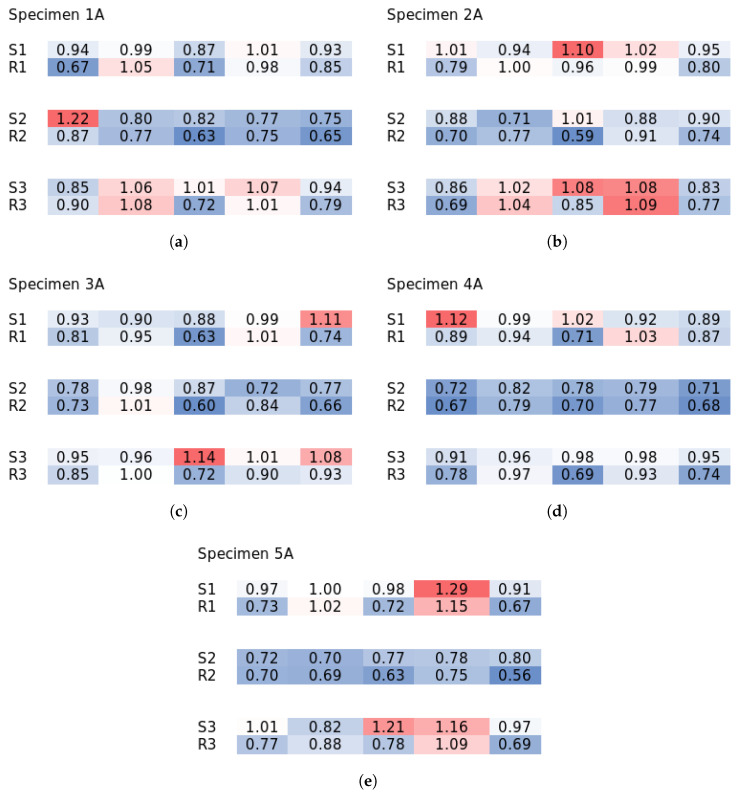
Results of the non-destructive orientation analysis, series A. Colors highlight deviations from 1.0. (**a**) Specimen 1A; (**b**) specimen 2A; (**c**) specimen 3A; (**d**) specimen 4A; (**e**) specimen 5A.

**Figure A7:**
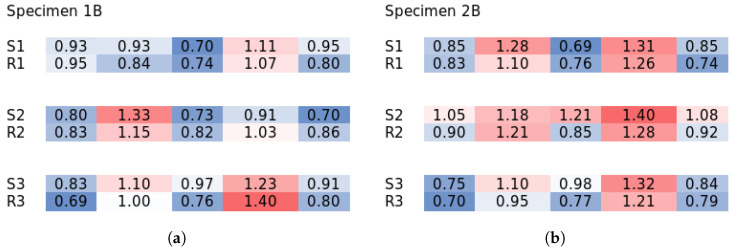

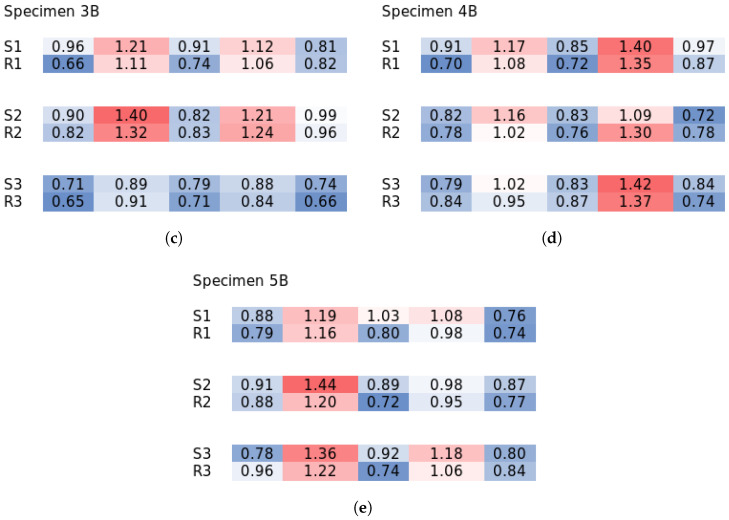
Results of the non-destructive orientation analysis, series B. Colors highlight deviations from 1.0. (**a**) Specimen 1B; (**b**) specimen 2B; (**c**) specimen 3B; (**d**) specimen 4B; (**e**) specimen 5B.

**Figure A8:**
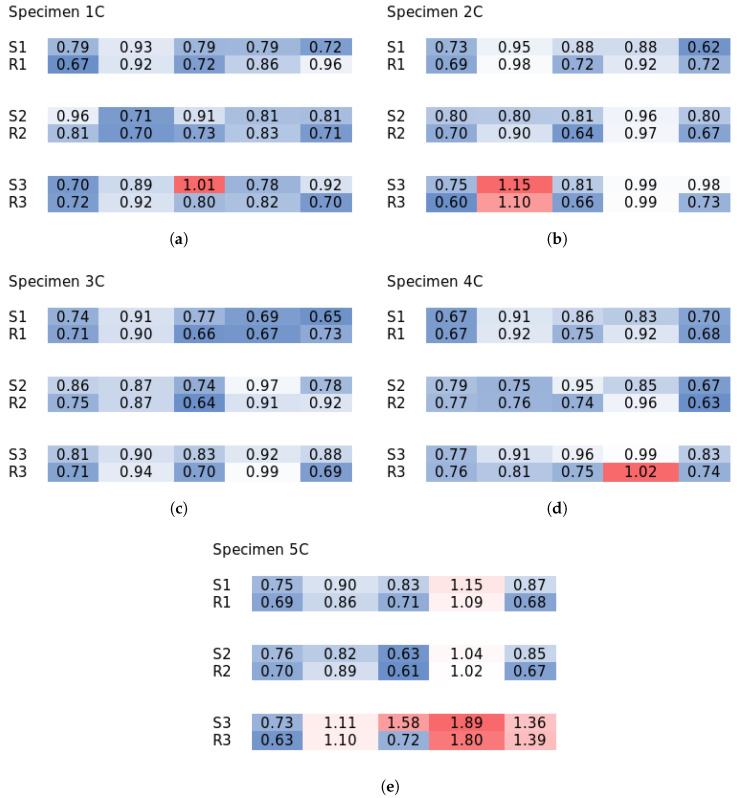
Results of the non-destructive orientation analysis, series C. Colors highlight deviations from 1.0. (**a**) Specimen 1C; (**b**) specimen 2C; (**c**) specimen 3C; (**d**) specimen 4C; (**e**) specimen 5C.

**Figure A9:**
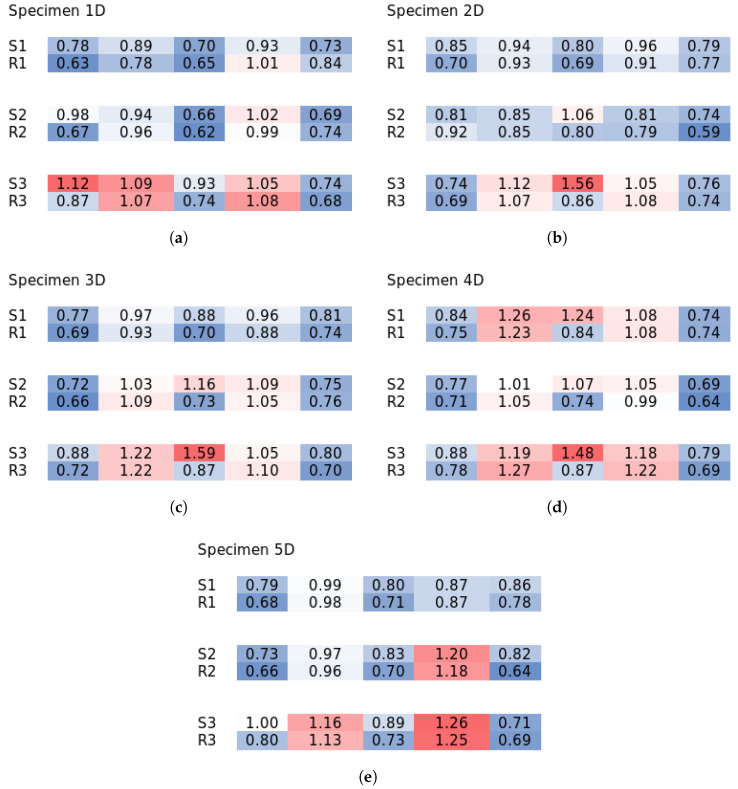
Results of the non-destructive orientation analysis, series D. Colors highlight deviations from 1.0. (**a**) Specimen 1D; (**b**) specimen 2D; (**c**) specimen 3D; (**d**) specimen 4D; (**e**) specimen 5D.

**Figure A10:**
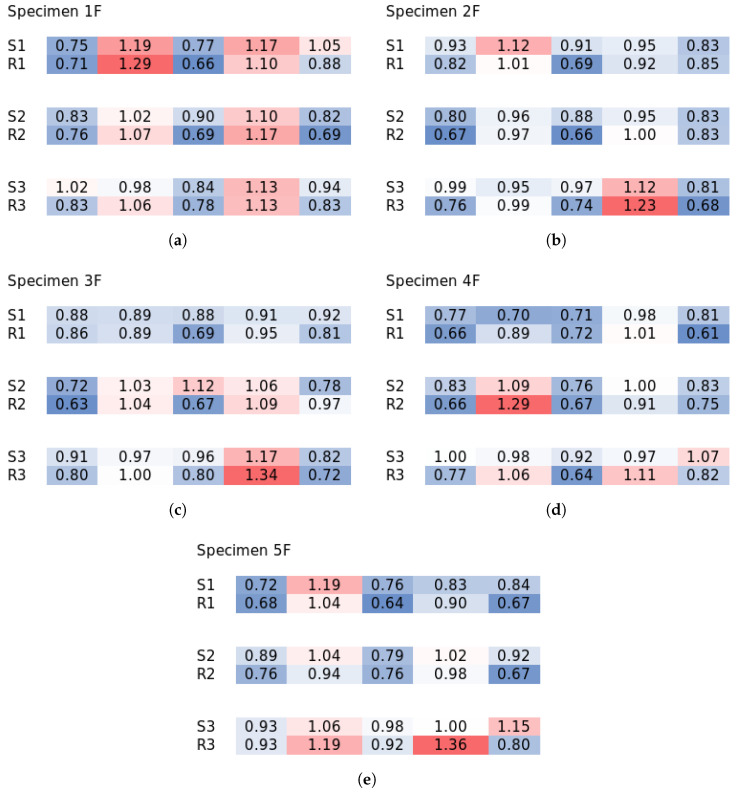
Results of the non-destructive orientation analysis, series F. Colors highlight deviations from 1.0. (**a**) Specimen 1F; (**b**) specimen 2F; (**c**) specimen 3F; (**d**) specimen 4F; (**e**) specimen 5F.

**Figure A11:**
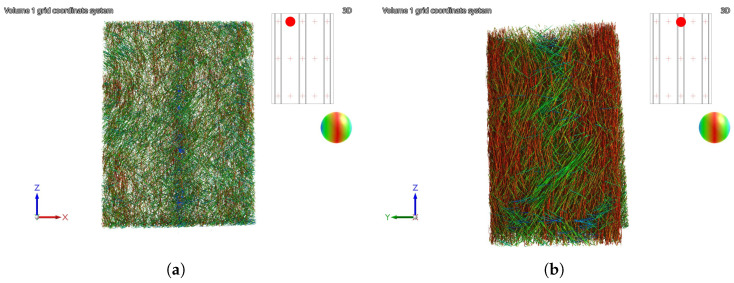

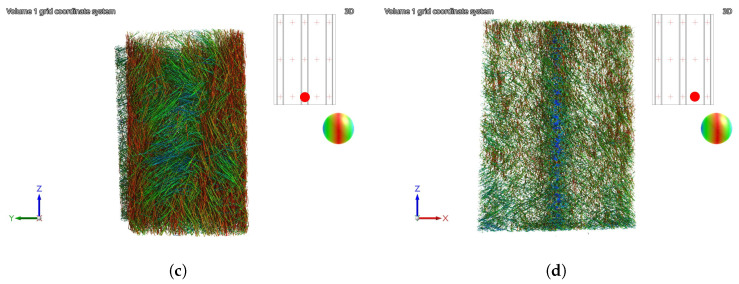
CT scans of specimen 1A from indicated positions in the top-right schematic. Colors indicate fiber orientations as per the colored ball on the right. Axis in the down-left corner is the display axis. Locations on specimen (**a**) row 1, column 2; (**b**) row 1, column 3; (**c**) row 3, column 3; (**d**) row 3, column 4.

**Figure A12:**
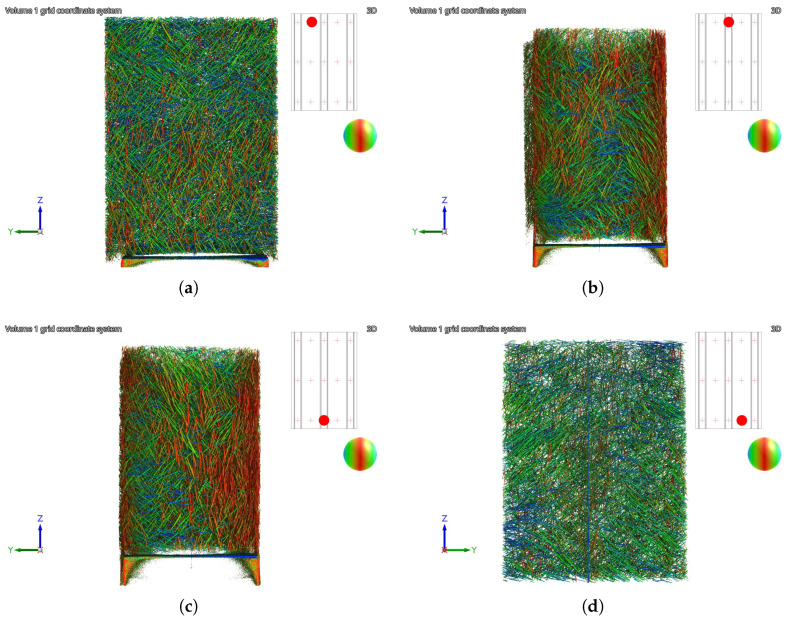
CT scans of specimen 1B from indicated positions in the top-right schematic. Colors indicate fiber orientations as per the colored ball on the right. Axis in the down-left corner is the display axis. Locations on specimen (**a**) row 1, column 2; (**b**) row 1, column 3; (**c**) row 3, column 3; (**d**) row 3, column 4.

**Figure A13:**
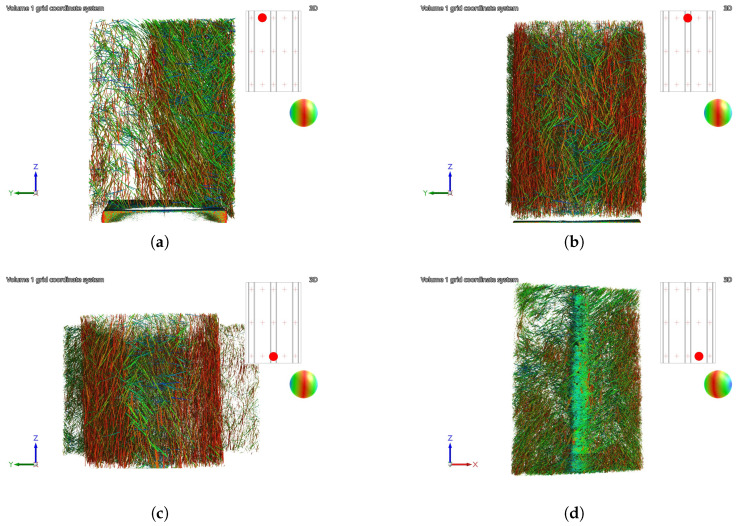
CT scans of specimen 2C from indicated positions in the top-right schematic. Colors indicate fiber orientations as per the colored ball on the right. Axis in the down-left corner is the display axis. Locations on specimen (**a**) row 1, column 2; (**b**) row 1, column 3; (**c**) row 3, column 3; (**d**) row 3, column 4.

**Figure A14:**
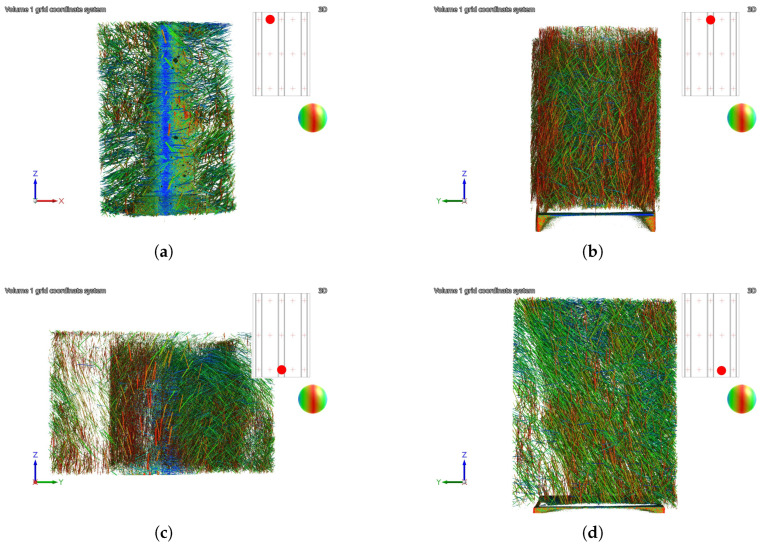
CT scans of specimen 4C from indicated positions in the top-right schematic. Colors indicate fiber orientations as per the colored ball on the right. Axis in the down-left corner is the display axis. Locations on specimen (**a**) row 1, column 2; (**b**) row 1, column 3; (**c**) row 3, column 3; (**d**) row 3, column 4.

**Figure A15:**
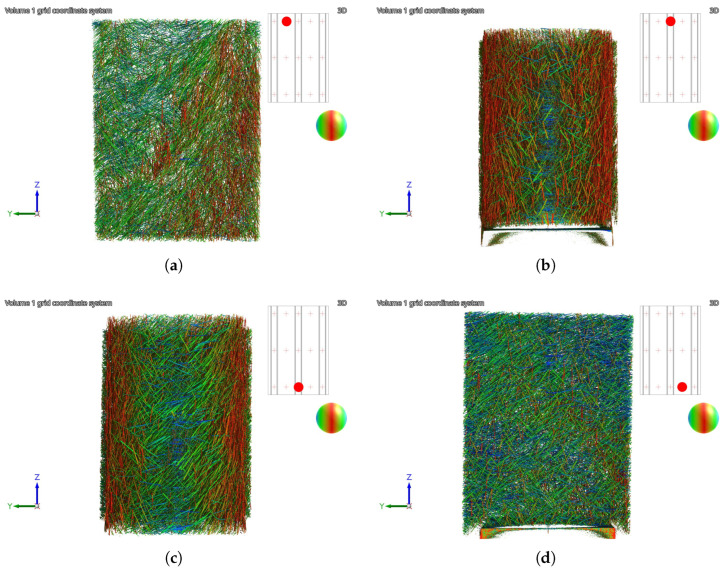
CT scans of specimen 5D from indicated positions in the top-right schematic. Colors indicate fiber orientations as per the colored ball on the right. Axis in the down-left corner is the display axis. Locations on specimen (**a**) row 1, column 2; (**b**) row 1, column 3; (**c**) row 3, column 3; (**d**) row 3, column 4.

## Data Availability

The original contributions presented in this study are included in the article. Further inquiries can be directed to the corresponding author.
